# Lumbar Radiofrequency Rhizotomy in Patients with Chronic Low Back Pain Increases the Diagnosis of Sacroiliac Joint Dysfunction in Subsequent Follow-Up Visits

**DOI:** 10.1155/2017/4830142

**Published:** 2017-02-01

**Authors:** Varun Kumar Rimmalapudi, Sanjeev Kumar

**Affiliations:** ^1^Department of Anesthesiology, University of Florida College of Medicine-Jacksonville, Jacksonville, Duval County, FL, USA; ^2^Department of Anesthesiology, University of Florida College of Medicine-Gainesville, Gainesville, Alachua County, FL, USA

## Abstract

Chronic back pain is often a result of coexisting pathologies; secondary causes of pain can become more apparent sources of pain once the primary pathology has been addressed. The objective of our study was to determine if there is an increase in diagnosis of Sacroiliac joint pain following a Lumbar Rhizotomy. A list of patients who underwent Lumbar Radiofrequency during a 6-month period in our clinic was generated. Records from subsequent clinic visits were reviewed to determine if a new diagnosis of SI joint pathology was made. In patients who underwent a recent Lumbar Rhizotomy procedure to treat facetogenic pain, the prevalence of Sacroiliac joint pain increased to 70%. We infer that there is a significant increase in the diagnosis of Sacroiliac joint syndrome following a Lumbar Rhizotomy, potentially due to unmasking of a preexisting condition. In patients presenting with persistent back pain after Lumbar Rhizotomy, the clinician must have a high degree of suspicion for latent Sacroiliac joint pain prior to attributing the pain to block failure. It would be prudent to use >80% relief of pain after a diagnostic medial branch block as a diagnostic criterion for facetogenic pain rather than the currently accepted >50% in order to minimize unmasking of preexisting subclinical pain from the SI joint.

## 1. Introduction

Low back pain is the most common pain in the modern society with estimates of lifetime prevalence as high as 84–90% [1] and the 5-year recurrence rate as high as 69% [2]. Multiple structures can contribute to lower back pain including but not limited to the lumbar vertebral bodies, intervertebral discs, facet joints, spinal nerves, the surrounding muscles, and ligaments. The incidence of Sacroiliac joint (SI joint) dysfunction in patients with back pain may range from 15 to 30% [3] and lumbar facet joints may account for 15 to 40% of back pain [4]. In about 2–10 percent of patients, the back pain becomes chronic in nature. A recent study estimated that almost 19% of adults in the US report persistent pain [5]. Sacroiliac joint pain and pain from facet joints have been shown to have a similar prevalence rates and occur in a similar patient population of older adults [6, 7]. The pain referral pattern from both these pains is also similar and a diligent physical examination is necessary to delineate the exact cause of back pain. In the subset of patients with chronic back pain, the pain is often a result of coexisting pathologies in multiple structures although the pain from one of them often dominates the clinical picture. The secondary causes of pain can become more apparent sources of pain to the patient once an intervention has been performed to target the primary cause. Our hypothesis was that subclinical Sacroiliac joint pain would become more apparent and is hence clinically diagnosed more often in the subsequent follow-up visits after an intervention such as radiofrequency rhizotomies of the medial branches of lower lumbar dorsal rami has been performed to address the pain from the lumbar facet joints.

## 2. Methods

Based on the number of Lumbar Radiofrequency rhizotomies performed at our institution within the study period, we estimated that we would reach a study population of 100 patients. In order to be able to account for our exclusion criteria, we calculated that a sample size of 50 patients would provide us a margin of error of 13.8% at a 95% confidence interval in determining the association of Sacroiliac joint dysfunction in clinic visits following a radiofrequency rhizotomy [8]. Approval was obtained from the institutional review board for a retrospective chart review (study number UF J 2015-30). The EPIC Ambulatory Database from the pain clinic at the University of Florida-Jacksonville was utilized and a report was generated including patients who underwent a radiofrequency rhizotomy of medial branches of dorsal rami of lumbar levels between 7/1/14 and 1/31/15. We included all patients who underwent the Lumbar Radiofrequency procedure at our institute during the study period and our exclusion criteria included patients who did not have at least 2 postprocedural clinic visits either due to lack of patient follow-up or due to the postprocedural visits being scheduled at a future time beyond our study period. A chart review was performed by the authors including the clinic visits for the procedural encounter and the 3 subsequent follow-up visits. Data collected included age, sex, and the presence or absence of Sacroiliac joint pain at the particular clinic visit. Presence or absence of Sacroiliac joint pain was assessed using physical examination findings including FABER/Patrick's test, Gaenslen's test, and the Fortin finger test (tenderness over area medial and inferior to posterior superior iliac spine), diagnostic assessment for the particular visit, and the procedure performed. We performed a literature search using the MEDLINE and EMBASE databases to identify the reported prevalence of different causes of low back pain. Of the available studies, we identified DePalma et al.'s study [9] as the one closest to our study population based on the fact that it included patients from a Chronic Pain Clinic with a similar male and female distribution ratio and a similar age group to our study population. Once the prevalence of Sacroiliac joint pain in the subsequent clinic visits after radiofrequency rhizotomy of medial branches of dorsal rami was determined, it was compared to the prevalence of Sacroiliac joint pain reported in DePalma et al.'s study [9]. IBM SPSS version 22 was used for the statistical analyses and a chi-square test was performed to compare the 2 patient groups.

## 3. Results

A total of 96 patients underwent the radiofrequency rhizotomy procedure during the study period. Of these, 46 patients were excluded, as they did not have the required minimum of 2 follow-up visits after the procedure. Of the 50 patients included in the study, 66 percent of the subjects were female and 34 percent were male. Age of the patients ranged from 34 to 84 with an average age of 57.8 years. DePalma et al.'s study population included patients referred to a Chronic Pain Clinic from various specialists and included patients with back pain for at least 12 months. The mean age of the included patients was 52.8 years and 65% of subjects were female [9].

**Figure 1:**
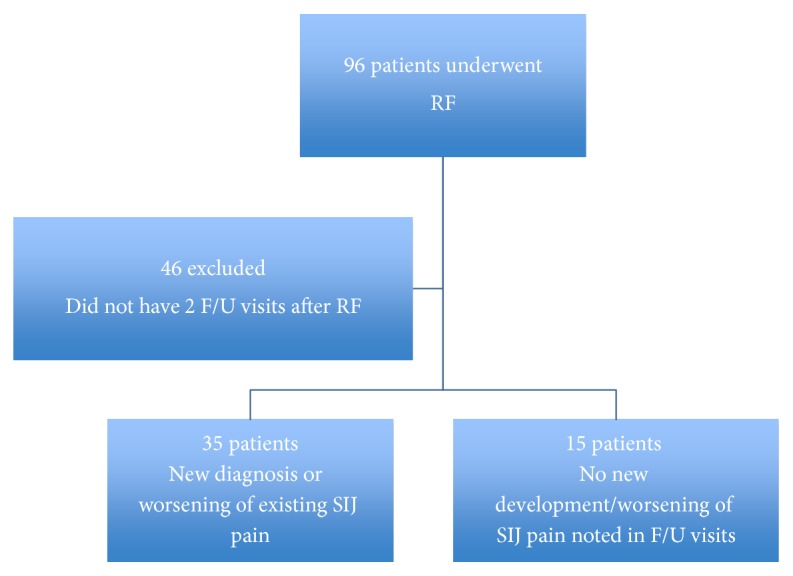
Study results depicting new development of Sacroiliac pain after Lumbar Radiofrequency rhizotomy. SIJ, Sacroiliac joint; RF, radiofrequency (lumbar) procedure.

Among the 50 patients, 35 patients developed Sacroiliac joint pain or had worsening of their preexisting SI joint pain (Figure 1). Of these 35 patients, 21 did not have signs of SI pain at their initial procedural visit and developed bilateral SI pain at the subsequent visits and 8 patients without prior signs of SI joint pain went on to develop unilateral SI joint pain at the subsequent visits. In 3 patients, unilateral Sacroiliac pain progressed to bilateral Sacroiliac pain in the subsequent follow-up visits and, in 3 patients, mild bilateral Sacroiliac pain at the initial procedural visit progressed to severe bilateral pain in the visits following the radiofrequency procedure. Among the 35 patients who had worsening of the Sacroiliac pain after the radiofrequency procedure, 23 were female and the rest were male.

**Figure 2:**
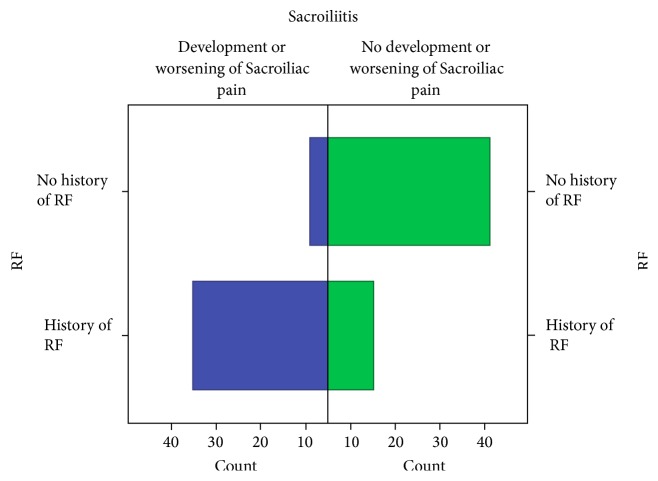
Incidence of Sacroiliac joint pain was 70% among patients with recent history of Lumbar Radiofrequency procedure compared to 18% among those without a recent history of the procedure.

The incidence of SI joint dysfunction was as high as 70% in our postrhizotomy sample but only 18.2% in DePalma et al.'s study of similar low back pain patients who did not undergo radiofrequency rhizotomy (Figure 2). A chi-square test was run using IBM SPSS version 22 to assess the association between Sacroiliac pain and history of a radiofrequency rhizotomy procedure. All the expected cell frequencies were greater than five. There was a statistically significant association between the diagnosis of Sacroiliac pain and the recent history of a radiofrequency rhizotomy procedure, *χ*^2^(1) = 27.435, *p* < 0.001. Phi (*φ*) and Cramer's *V* were also calculated to assess the strength of association between the variables of Sacroiliac pain and a recent history of radiofrequency rhizotomy (Table 2). All the expected frequencies were greater than five and a strong association was noted between the variables, *φ* = 0.524, *p* < 0.001 (Table 1).

## 4. Discussion

Several factors have been associated with the development of Sacroiliac joint pain including injury from accidents and falls, gait abnormalities, pregnancy, leg length discrepancies, scoliosis, ankylosing spondylitis, psoriatic arthritis, and previous lumbar fusion surgery [3, 10–12]. After a review of the available literature to date, we could not find any study that showed a correlation between development of Sacroiliac joint pain and a history of Lumbar Radiofrequency rhizotomy procedure. Our study found a statistically significant association between these variables. Several factors could be responsible for this finding, the most plausible being the change in gait or walking pattern of the patient after the pain from the lumbar zygapophyseal joints is relieved by the radiofrequency procedure. Since coexistence of pathologies is extremely common among patients with chronic back pain, it is also possible that after the primary cause of back pain has been addressed using an interventional approach, the patient perceives the preexisting Sacroiliac dysfunction more prominently.

Our study suffered from several limitations. Firstly, our study population was limited by our relatively recent transition to the EPIC system in our clinic. Due to the time frame of our data collection of 6 months, several of the patients could not complete the required minimum of 2 postprocedural follow-up visits, decreasing our study population to 50. Although the primary reason for lack of follow-up was the inability to make appointments within the time frame of our data collection, the lack of pain after the procedure cannot be ruled out as the reason for patients not following up. Secondly, our control group was chosen from a different study. DePalma et al. utilized multiple interventions including discography, diagnostic Sacroiliac joint injections, and interspinal ligament injections in order to delineate and determine the cause of back pain in a sample of patients presenting to a Chronic Pain Clinic, while we relied on physical examination findings alone to diagnose Sacroiliac pain in patients presenting for follow-up visits after a Lumbar Rhizotomy performed for facetogenic pain. Although we made efforts to minimize the differences between the demographics and characteristics of both groups, it would have been ideal to choose the groups from the same clinic. We did start this endeavor and such study is underway in our clinic currently. Larger studies are certainly needed to shed further light on this interesting phenomenon.

In summary, our study brings up three important points. Firstly, in patients presenting with persistent low back pain after having a lumbar medial branch radiofrequency ablation, the clinician must have a high degree of suspicion for Sacroiliac joint pain prior to attributing the pain to block failure. Secondly, it emphasizes the importance of a comprehensive physical examination in any patient presenting with lower back pain. Provocative Sacroiliac joint maneuvers such as FABER-Patrick's test and Gaenslen's test must always be performed during the pre- and postprocedural clinic visits in patients with lumbar zygapophyseal joint pain. These physical examination maneuvers should ideally be followed by confirmatory diagnostic joint blocks prior to therapeutic measures. Thirdly, it highlights the importance of clearly defining the index pain and utilizing a cut-off of 80–100% reduction in pain after a diagnostic facet block as utilizing a conventional >50% pain relief may result in residual pain from a preexisting chronic condition that may become unmasked after the Lumbar Rhizotomy procedure.

## Figures and Tables

**Table 1:** Chi-square tests. Chi-square tests were computed for a 2 × 2 table; 0% of the cells had an expected count less than 5. Minimum expected count was 22.

Measure	Value	df	Asymptomatic significance (2-sided) *p*
Pearson's chi-square	27.435	1	<0.001
Continuity correlation	25.365	1	<0.001
Likelihood ratio	28.960	1	<0.001
Linear by linear association	27.161	1	<0.001

**Table 2:** Symmetric measures. Phi and Cramer's *V* were calculated to assess strength of association between the two dichotomous variables, Sacroiliac joint dysfunction, and history of Lumbar Radiofrequency.

Measure (nominal by nominal)	Value	Approximate significance (*p*)
Phi	0.524	<0.001
Cramer's *V*	0.524	<0.001
